# Identifying Chronic Lymphocytic Leukaemia and Small Lymphocytic Lymphoma: An Uncommon Case of Ocular Adnexal Lymphoma

**DOI:** 10.7759/cureus.103586

**Published:** 2026-02-14

**Authors:** Sue Ann Chang, Xiong Khee Cheong, Juen Kiem Tan, Sivakumar Palaniappan, Alia Suzana Asri, Nor Rafeah Tumian, Nordashima Abd Shukor, Chia Yin Chong, Othmaliza O, S Fadilah Abdul Wahid

**Affiliations:** 1 Department of Medicine, Faculty of Medicine, Universiti Kebangsaan Malaysia, Kuala Lumpur, MYS; 2 Department of Internal Medicine, Hospital Canselor Tuanku Muhriz, Universiti Kebangsaan Malaysia, Kuala Lumpur, MYS; 3 Department of Haematology, Hospital Canselor Tuanku Muhriz, Universiti Kebangsaan Malaysia, Kuala Lumpur, MYS; 4 Department of Pathology, Faculty of Medicine, Universiti Kebangsaan Malaysia, Kuala Lumpur, MYS; 5 Department of Pathology, Hospital Canselor Tuanku Muhriz, Universiti Kebangsaan Malaysia, Kuala Lumpur, MYS; 6 Department of Radiology, Faculty of Medicine, Universiti Kebangsaan Malaysia, Kuala Lumpur, MYS; 7 Department of Radiology, Hospital Canselor Tuanku Muhriz, Universiti Kebangsaan Malaysia, Kuala Lumpur, MYS; 8 Department of Ophthalmology, Faculty of Medicine, Universiti Kebangsaan Malaysia, Kuala Lumpur, MYS; 9 Department of Ophthalmology, Hospital Canselor Tuanku Muhriz, Universiti Kebangsaan Malaysia, Kuala Lumpur, MYS

**Keywords:** chronic lymphocytic leukemia, cll, ocular adnexal lymphoma, sll, small lymphocytic lymphoma

## Abstract

Chronic lymphocytic leukaemia (CLL) and small lymphocytic lymphoma (SLL) are typically classified and treated as different manifestations of the same disease. Compared to the Western hemisphere, this type of lymphoma is rare in Asian countries. They usually manifest as anaemia, organomegaly, or lymphadenopathy. Ocular adnexal lymphoma (OAL) is a rare presentation of lymphoma that can affect any part of the orbital region. We present a gentleman who sought treatment at our centre for this rare malignancy, with an atypical presentation of painless swelling over the upper eyelid. The initial diagnosis of hematoma secondary to trauma was swiftly discarded following surgery, revealing a lobulated mass. Subsequently, histopathological examination points towards a diagnosis of CLL/SLL. We illustrate the importance of multidisciplinary management with several departments, striving to achieve the best possible outcome.

## Introduction

Chronic lymphocytic leukaemia (CLL) and small lymphocytic lymphoma (SLL) are essentially the same disease, but with different manifestations, and are managed similarly [1]. They are an indolent form of neoplasm comprising functionally incompetent, mature B-cell lymphocytes and characterized by progressive accumulation of these cells in peripheral blood, bone marrow, and lymphoid tissues [2,3]. In CLL, these abnormal lymphocytes are seen in large numbers (5x10^9^/L or more) in blood, bone marrow, and lymphoid tissue. On the other hand, SLL presents with infiltration of CLL cells in tissues, leading to organomegaly (e.g. hepatosplenomegaly) or lymphadenopathy with monoclonal B lymphocytes less than 5x10^9^/L in peripheral blood [4]. Therefore, more emphasis is placed on obtaining tissue biopsies for a definitive diagnosis, as with any other form of lymphoma. Only a few reports have described both CLL and SLL separately, and most treat them as similar entities with the World Health Organization (WHO) grouping both in the same classification of mature B-cell neoplasms [5,6]. Ocular adnexal lymphoma (OAL) is an uncommon presentation of lymphoma and may involve the conjunctiva, orbital soft tissue, eyelid, or lacrimal glands [7]. We present a gentleman with an atypical presentation of a rare form of lymphoma requiring a multidisciplinary approach. Due to the history of presentation and radiological finding, he was initially thought to have a benign condition. Subsequently diagnosis was made through histopathological findings.

## Case presentation

A 60-year-old gentleman with a background of hypertension presented to our center with the chief complaint of left eye swelling after sustaining trauma while closing his car boot. Initially, it was a small, painless lump that gradually increased in size over the course of a month. Subsequently, it rapidly increased in size over two weeks, prompting him to seek treatment. There was no blurring of vision or eye discharge, no constitutional or systemic symptoms. He has no family history of malignancy and is a retiree.

**Figure 1:**
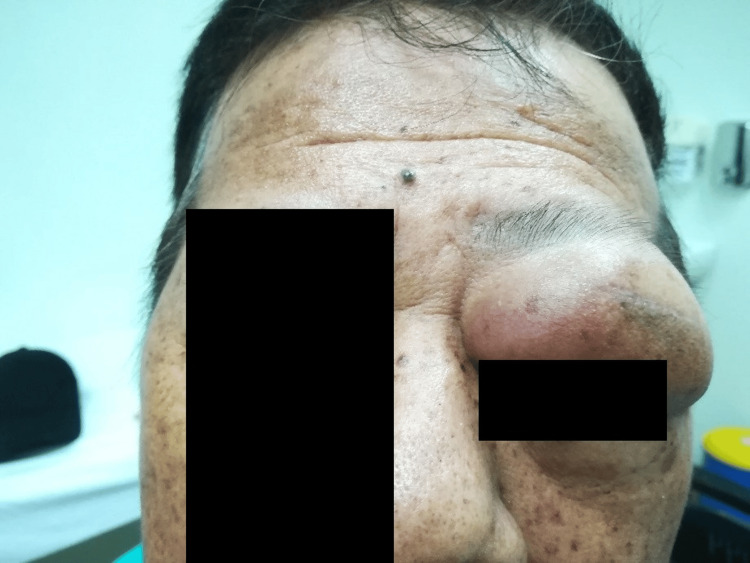
Left upper eyelid swelling

**Figure 2:**
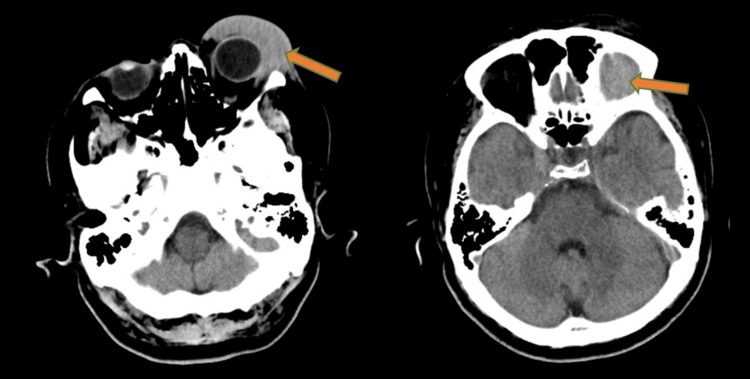
Plain CT orbit; axial cut showing hyperdensity at the left periorbital and superior extraconal regions extending to orbital apex measuring 4.5x3.8x3.2cm (orange arrow)

He was seen by the ophthalmology team, who admitted him after complaints of increasing painless swelling and diplopia as his eyelid aperture was narrowing. Clinically, the swelling over the left upper eyelid was firm and mobile with overlying erythema (Figure 1). Additionally, there was mild proptosis and chemosis with diplopia on vertical gaze. However, there were no cranial nerve defects, and extraocular movements were unhindered. A plain computed tomography (CT) of the brain/orbit and facial bones demonstrated a hyperdense lesion at the left periorbital and superior extraconal regions measuring 4.5x3.8x3.2cm in size, likely to represent a haematoma with attenuation at 46-55HU extending to the orbital apex (Figure 2). Otherwise, there were no focal cortical lesions.

His complete blood count shows a hypochromic microcytic type of anaemia (haemoglobin (Hb) 11.2g/dL; mean cell volume 73 fL; mean corpuscular haemoglobin (MCH) 23.6pg) with mild lymphocytosis (white blood cell count (WBC) 7.0 x10^9^/L; lymphocytes 4.1x10^9^/L) but otherwise average platelet counts. He had mild renal impairment (creatinine 126 μmol/L) and normal lactate dehydrogenase (173 U/L). The results as shown in Table 1.

**Table 1:** Blood investigation results

Test	Result	Unit	Normal Range
Haemoglobin	11.2	g/dL	12-15
Mean cell volume	73	fL	83-101
Mean corpuscular haemoglobin	23.6	pg	27-32
White cell count	7.0	x10^9^/L	4-10
Lymphocyte count	4.1	x 10^9^/L	1-3
Platelet	149	x 10^9^/L	150-410
Urea	5.2	mmol/L	3.2-7.4
Creatinine	126	µmol/L	63.6-110.5
Lactate dehydrogenase	173	U/L	125-220

Given a history of trauma, CT finding of a hyperdense lesion with attenuation of between 30 and 70 HU, and mild hypochromic microcytic anaemia on blood investigation, the diagnosis of haematoma is likely. Other differential diagnoses to consider for a unilateral painless eye swelling are chalazion, cellulitis, contact dermatitis, and orbital tumour.

**Figure 3:**
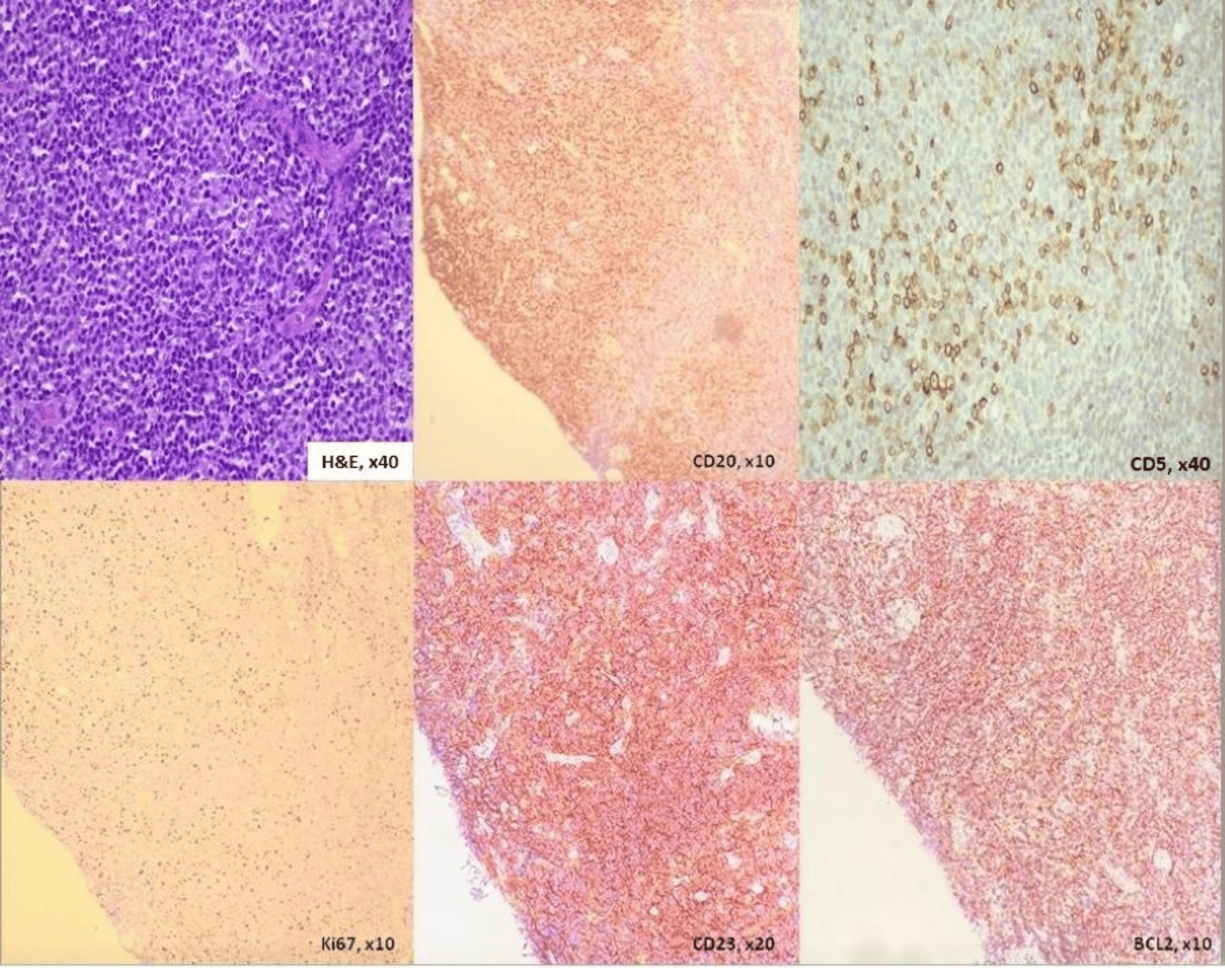
Eyelid mass biopsy showing diffuse infiltration by small, uniform neoplastic lymphoid cells. Immunohistochemical study shows positivity for CD20, CD5 (weak and scattered), CD23, and BCL-2. The Ki67 immunostain shows a proliferative index of 10%-20%.

He was scheduled for needle aspiration of his left eye swelling under general anaesthesia. However, they could not aspirate any blood and proceeded to incise the overlying skin of the eyelid, revealing a pinkish-lobulated mass. The histopathological examination of the sample revealed neoplastic lymphoid cells that were positive for CD20, CD79a, CD43, CD23, BCL-2, and CD5, and negative for CD3, cyclin D1, and CD10. The Ki-67 proliferative index was 10%-20% with a higher index in the pseudofollicular area (Figure 3). There was evidence of Kappa light chain restriction, and the findings suggest chronic lymphocytic lymphoma/small lymphocytic lymphoma. CT staging revealed multiple lung nodules with spiculated margins, hepatomegaly measuring 20 cm, and enlarged lymph nodes in the cervical, supraclavicular, mediastinal, porta hepatic, aortocaval, para-aortic, and bilateral inguinal regions, with the largest measuring 11 mm.

A bone marrow aspirate revealed hypercellular marrow with lymphocytosis, and trephine showed diffuse infiltration of small monomorphic lymphoid cells at intertrabeculae and some paratrabeculae areas positive for CD20, CD5, and CD23; CD3 showed scattered reactive T cells. Immunophenotyping revealed abnormal lymphoid cell population gated at CD45 bright region with low side scatter, and population gated at CD19 positive cells are positive for CD20, CD22, CD5, CD23, CD25, and CD200 with kappa light chain restriction. The presence of markers, especially CD5, CD23, CD20, CD 200 with CD10, and cyclin D1, points towards the diagnosis of CLL. Cytogenetics were normal with fluorescence in situ hybridization (FISH) negative for p53, ATM, D13S319, 13q34, and CEP12 deletion. This shows a favourable prognosis for him.

He was diagnosed with chronic lymphocytic leukaemia (CLL)/small lymphocytic lymphoma (SLL); Rai stage II/Binet stage B [8,9]. In retrospect, the fast-growing nature, firm, and salmon pink appearance of the mass would suggest the diagnosis of ocular adnexal lymphoma. He was started on chemotherapy with rituximab, fludarabine, and cyclophosphamide after a pre-chemotherapy workup showed normal ejection fraction and viral screening was non-reactive. He has since completed his first cycle without any complications and is awaiting his second cycle of chemotherapy.

## Discussion

A literature search on prevalence shows a high proportion of CLL/SLL cases in the Western Hemisphere as opposed to Asia [10]. A large-scale study in the United States of 596,476 patients with lymphoma found that 18.6% of them were CLL/SLL, while Japanese and Korean studies with 9424 patients and 5318 patients, respectively, showed an incidence of 1.11% and 1.8%, respectively [11-13]. Similarly, in South East Asia, a study in Thailand on 4056 cases showed only 1.5% of cases were CLL/SLL, and a previous study two decades ago by our institution’s pathology department did not identify any case of CLL/SLL among 49 lymphoma slides, underlying its rarity in this region [14]. Ocular adnexal lymphoma (OAL) represents the most common primary orbital malignancy, with as high as 55% of cases [15]. However, its incidence is estimated to be as low as 0.2 per 100,000 individuals.

CLL/SLL patients may present with lymphadenopathy, organomegaly, pallor, and petechial rashes. However, as many as 25%-50% may present with incidental findings on routine blood work [16]. Despite large numbers of leukocytes in CLL, the signs and complications of leukostasis are rarely seen due to the small size of mature lymphocytes [2]. In the case of OALs, they present insidiously as a slowly progressive firm, palpable mass commonly seen between the fifth and seventh decades of life. It may be associated with pain, swelling, diplopia, or reduced vision. Clinically, it is seen as a salmon-pink patch for conjunctival lymphoma [7]. A systematic review showed 25.6% of patients' first manifestation of CLL was ophthalmic symptoms [17]. Most of the cases reported presented with similar presentation as mentioned above and were diagnosed by histopathology. One case series noted development of OAL among patients with history of CLL [18].

As mentioned, to diagnose CLL, more than 5x10^9^/L monoclonal B-lymphocytes in the peripheral blood and clonality of B-cells confirmed by flow cytometry should be observed [4]. Flow cytometry via peripheral blood is sufficient, therefore obviating the need for bone marrow biopsy. Typical immunophenotype for CLL/SLL are CD5+, CD23+, CD10-, CD19+, CD20 (+low), CD 22 (+low), FMC7-, CD79b (+low) [19]. Cytogenetic abnormalities detected via FISH can also help in further management and prognostication (e.g., trisomy 12). CT scans aid in staging and subsequently in monitoring disease progression and response. PET/CT is generally not useful due to poor uptake of radiotracers in this indolent type of malignancy. However, it may be performed if clinical indicators suggest transformation, second malignancies, or severe infections.

Staging for CLL relies on Rai and Binet systems (Table 2), developed in the 1970s and 1980s, respectively [8,9].

**Table 2:** Chronic lymphocytic leukaemia (CLL) staging

Rai system	
0	Lymphocytosis (lymphocytes ≥ 15x10^9^/L in blood + ≥40% lymphocytes in bone marrow)
I	Lymphocytosis + lymphadenopathy
II	Lymphocytosis + splenomegaly and/or hepatomegaly ± lymphadenopathy
III	Lymphocytosis + anaemia (haemoglobin <11 g/dL/haematocrit <33%) ± lymphadenopathy/splenomegaly/hepatomegaly
IV	Lymphocytosis + thrombocytopaenia (platelets <100x10^9^/L) ± anaemia/lymphadenopathy/splenomegaly/hepatomegaly
Binet system	
A	Haemoglobin ≥10 g/dL + platelet ≥100x10^9^/L+ <3 enlarged areas
B	Haemoglobin ≥10 g/dL + platelet ≥100x10^9^/L+ ≥3 enlarged areas
C	Haemoglobin <10 g/dL ± platelet <100x10^9^/L+ any number of enlarged areas

SLL utilizes the Lugano Modification of Ann Arbor staging (Table 3). The criteria of staging are similar to the Ann Arbor staging, with the addition that nodes above the diaphragm with splenic involvement are considered stage III. Also, a single extranodal lesion without nodal involvement is stage I, while those with limited, contiguous extranodal involvement are stage II [20].

**Table 3:** Small lymphocytic lymphoma (SLL) staging *Whether stage II bulky disease is treated as limited or advanced disease may be determined by histology and prognostic factors.

Stage	Involvement	Extranodal Status
Limited		
I	One node or a group of adjacent nodes	Single extranodal lesion without nodal involvement
II	≥2 nodal groups on the same side of the diaphragm	Stage I or II by nodal extent with limited contiguous extranodal involvement
II bulky*	II as above with “bulky” disease	Not applicable
Advanced		
III	Nodes on both sides of the diaphragm; nodes above the diaphragm with spleen involvement	Not applicable
IV	Additional noncontiguous extralymphatic involvement	Not applicable

While the prognosis for CLL/SLL depends on TP53 mutation, the presence of del(11q) or del(17p) or three or more unrelated chromosomal abnormalities in more than one cell on karyotyping, and the site of involvement at the eyelid for OALs, suggests a more aggressive tumour. Bilateral presentation and/or clinical symptoms on presentation are associated with a poorer prognosis.

According to the National Comprehensive Cancer Network (NCCN) guidelines, all patients without symptoms of disease may be observed during follow-ups. Otherwise, treatment of CLL/SLL largely depends on two aspects: if the patient is aged above 65 years and/or has significant comorbidities, and if there is a TP53 mutation/del(17p). The preferred therapy for all conditions is a covalent Bruton’s tyrosine kinase (BTK) inhibitor or venetoclax-based combination regimens. Other suggested regimes include fludarabine, cyclophosphamide, and rituximab (FCR), as in our case [21]. Regarding OALs, radiotherapy, monoclonal antibody treatment, and radioimmunotherapy may be offered in addition to conventional chemotherapy [22].

## Conclusions

CLL/SLL is a rare malignancy in this region, coupled with an uncommon site involving the ocular adnexa. This case report underlines the importance of a multidisciplinary approach in management; the sample obtained by the ophthalmology team, the histopathological examination by the pathology unit, subsequent staging, and the initiation of treatment by the haematology unit. In addition to support groups and non-governmental organisations, the patient was promptly and adequately treated, improving his prognosis and overall survival.
